# Determination of uranium reference levels in the urine of Warsaw residents (Poland)

**DOI:** 10.1007/s10967-014-3787-5

**Published:** 2014-12-20

**Authors:** E. Starościak, L. Rosiak

**Affiliations:** Central Laboratory for Radiological Protection, Konwaliowa 7, 03-194 Warsaw, Poland

**Keywords:** ^238^U, ^234^U, Urine, Alpha spectrometry

## Abstract

This paper presents the results of determination of activity concentration of ^238^U and ^234^U in urine samples of people living in Warsaw (capital of Poland) to evaluate background level of these radionuclides excretion rate (mBq day^−1^). The samples were taken from women and men in age between 3 and 97 years. The results obtained for ^238^U daily excretion rate were in range 0.44–30.54 mBq day^−1^ and for ^234^U in range 0.33–28.61 mBq day^−1^. 70 % results were below 6 mBq day^−1^ upper limit recommended by ICRP for non-exposed people (ICRP, ICRP publications No. 23, report of the task group on reference man, 1975). The results are discussed and some conclusions based upon average values were drawn.

## Introduction

Uranium is primordial radioactive element ubiquitously present in the earth’s crust. It is found in all components of the environment. Uranium can reach the human body mostly by ingestion of drinking water and foodstuffs [1]. Uranium and its compounds are highly toxic both from chemical and radiological standpoints. Levels of ^238^U and ^234^U in food products in Poland were determined by Pietrzak-Flis et al. [2, 3]. The highest activity concentrations of ^238^U and ^234^U were found in green vegetables and in certain species of fish. Uranium is poorly absorbed by the digestive track and most of the absorbed uranium is eliminated with the urine within a short time. Uranium excretion rate in urine is proportional to the uranium level in the body [4]. Thus urine sample is the best biomarker for the detection of uranium intake.

In connection with cases of illegal turnover of fissile materials and more and more frequent using of depleted uranium in military and civilian sector exists a danger of absorbing these elements by inhalation and ingestion way in case of radiation incident. This exposure concerns not only workers and services, but also people of population—inhabitants of region surrounding. However, many factors may effect the ^238^U and ^234^U intake and lead to the variation in the body burden in different humans. Due to the large variance in the estimates of ^238^U and ^234^U content among different populations, the prospective of this article is limited to the evaluation of the reference level of these radionuclides excretion rate in the urine samples of people living in capital city of Poland–Warsaw. We also want to compare our results with data reported by other authors.

For our purpose, to evaluate the low exposure of the members of the public, a sensitive method is needed. The method used has been adapted from one that is in routine operational use for measurements on environmental samples, e.g. food, water, soil [5].

## Experimental

### Sample collection

Samples were collected from 84 volunteers of both sex who had never worked with uranium compounds. Volunteers were informed about the aim of this work and promised to collect an urine sample according to guidelines provided by an urologist (day and night samples: first day—all portions of urine without the first morning one, second day—only first morning urine). 41 females and 43 males participated in this study. Their ages ranged from 3 to 97 years. All people were inhabitants of Warsaw.

### Standard reference material

^232^U standard reference material, purchased from National Institute of Standards & Technology, USA, under the code number 4324B, was diluted and used as spiking tracer for analyzing uranium.

### Pretreatment

The 24 h urine samples were collected in a 3 l plastic boxes. Volume of each sample was measured by calibrated cylinders. The sample was carefully stirred and 1 l was taken for analysis. The volume of the sample was in range 0.22–2.92 l day^−1^. After addition to the sample of urine a known quantity of ^ 232^U as internal standard yield tracer and 50 ml concentrated HNO_3_, the sample was heated on a hot plate at 100 °C and evaporated almost to dryness. The residue was dissolved in 8 M HNO_3_.

### Radioanalytical method

Radiochemical method of uranium determination consists in separation of its isotopes from thorium isotopes by ion exchange on chromatography column. Thorium is deposited on the column and uranium passes to the solution. Next, uranium was extracted with the solution of tributylphosphate in kerosene, then reextracted into the aqueous phase. After evaporation of the extract, with addition of concentrated sulfuric acid electrodeposition on stainless steel discs was carried out. Uranium activity was measured by alpha spectrometry system with PIPS detectors, (Canberra, USA) for 250,000 s. The mean counting efficiency was 32 % and the background was for ^238^U 2.4 × 10^−5^ s^−1^ and for ^234^U 4.4 × 10^−5^ s^−1^ in their energy regions. The mean chemical yield for uranium was 63 % and the minimal detection activity was 0.5 mBq l^−1^. The uncertainties given with the final results are the combined uncertainties of the whole process of analysis. Uncertainties of presented results did not exceed 10 % of the obtained values.

### Analytical quality control

Participation in an international Radiotoxicology Intercomparison PROCORAD 2012 on the determination of uranium in urine samples. Emphasis in this proficiency test was placed on both the accuracy and the evaluation of analytical uncertainty. In the final evaluation, both scores for trueness and precision were combined. A result was defined “acceptable” when it obtained “acceptable” score in both criteria. Table 1 shows the results.

**Table 1:** Results on the determination of ^238^U and ^234^U in urine samples—PROCORAD 2012

Sample	Isotope	Procorad value [Bq per sample]	Procorad reference value [Bq per sample]		Procorad Unc. [Bq per sample]	Lab. value [Bq per sample]	Lab. Unc. [Bq per sample]	Final score
			Minimal	Maximum				
A	^238^U	1.74 × 10^−1^	1.40 × 10^−1^	2.17 × 10^−1^	5.00 × 10^−3^	1.67 × 10^−1^	1.30 × 10^−2^	Accept
A	^234^U	1.68 × 10^−1^	1.12 × 10^−1^	2.07 × 10^−1^	5.00 × 10^−3^	1.70 × 10^−1^	1.30 × 10^−2^	Accept
B	^238^U	3.56 × 10^−2^	2.74 × 10^−2^	4.41 × 10^−2^	1.00 × 10^−3^	3.80 × 10^−2^	2.00 × 10^−3^	Accept
B	^234^U	3.45 × 10^−2^	2.50 × 10^−2^	4.47 × 10^−2^	1.20 × 10^−3^	3.60 × 10^−2^	2.00 × 10^−3^	Accept

## Results and discussion

**Fig. 1:**
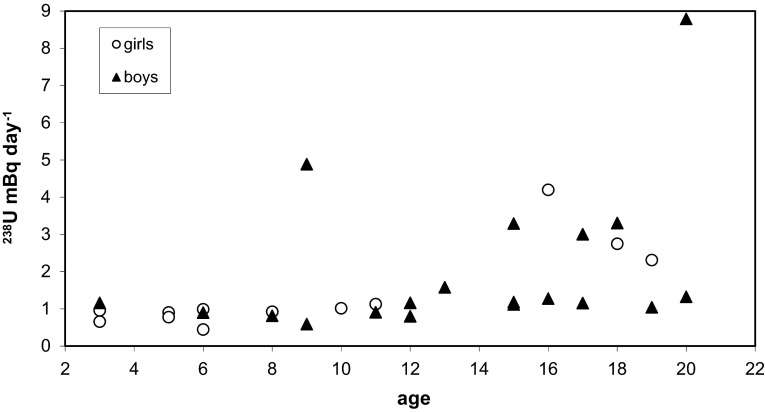
^238^U excretion rate in urine for girls and boys (3–20 years old)

**Fig. 2:**
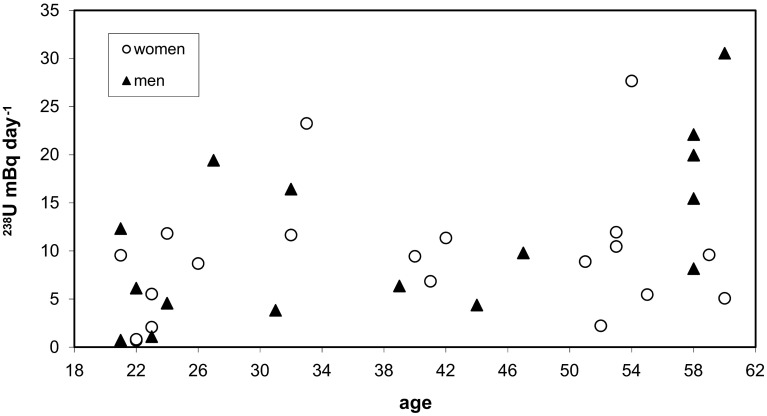
^238^U excretion rate in urine for females and males (21–60 years old)

**Fig. 3:**
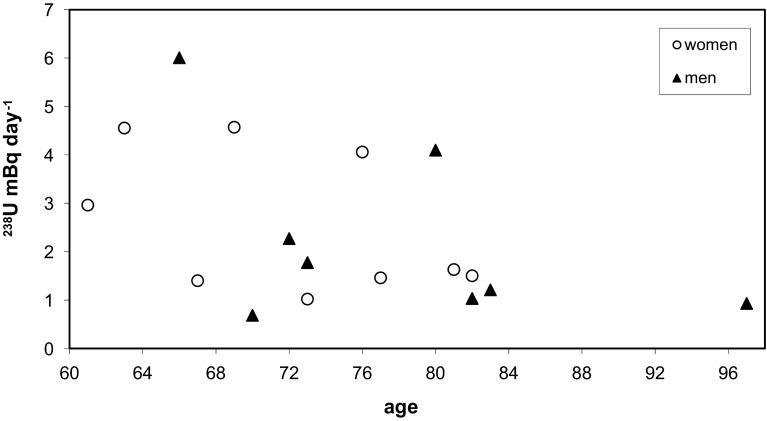
^238^U excretion rate in urine for females and males (61–97 years old)

The final results are reported as excretion rate (mBq day^−1^) obtained by multiplying uranium activity concentration (mBq l^−1^) by the volume of urine excreted in a day. The results of ^238^U excretion rate in urine are illustrated in Figs. 1, 2 and 3. Figures 1 and 2 shows ^238^U excretion rate in urine for girls and boys in age from 3 to 20 years. The results range was between 0.44 ± 0.04 and 8.79 ± 0.77 mBq day^−1^. Mean excretion rate for children in age 3–20 years old was 1.42 ± 1.10 (median 0.97) for girls and 2.01 ± 2.0 (median 1.16) mBq day^−1^ for boys. About 80 % of results oscillated from 0.44 to 1.58 mBq day^−1^. The mean value of all sample of this age class resulted 1.78 ± 1.71 (median 1.13) mBq day^−1^. Our results are very similar to those obtained by Al-Jundi et al. [6] from Jordania: 0.46–8.053 mBq day^−1^. In this age group low correlation (*R* ^2^ = 0.23) between excretion rate and age was observed.

In second age group from 21 to 60 years old people values of excretion rate of ^238^U were higher than for children. The results range was between 0.70 ± 0.06 and 30.54 ± 3.18 mBq day^−1^. Mean excretion rate for women was 9.15 ± 6.72 (median 9.17) and for men was 11.33 ± 8.29 (median 8.98) mBq day^−1^. The mean value of all samples in this age class was 10.12 ± 7.56 (median 9.17) mBq day^−1^. Al-Jundi et al. [6] gave range beetwen 0.226 and 42.490 mBq day^−1^. Observed correlation (*R* ^2^ = 0.17) between excretion rate and age in this age group was similar to this for children.

Figure 3 illustrates ^238^U excretion rate in urine for women and men in age 61–97 years. In this age group for ^238^U we observed decrease of excretion rate values. It may be due to a slower metabolism in the elderly and less consumption of food and beverages. The results ranged between 0.68 ± 0.05 and 6.01 ± 0.58 mBq day^−1^. Mean excretion rate for old women was 2.57 ± 1.47 (median 1.63) and for old men was 2.25 ± 1.87 (median 1.49) mBq day^−1^. The mean value of all samples in this age group was 2.42 ± 1.63 (median 1.63) mBq day^−1^. In age group above 60 years Al-Jundi et al. [6] observed 0.491–9.168 mBq day^−1^. In this age group inversely proportional relationship (*R* ^2^ = 0.23) between excretion rate and age was observed.

Mean value of all samples was 5.49 ± 6.48 (median 2.85) mBq day^−1^. No correlation between the daily excretion of ^238^U and sex of individuals was found. Data obtained for ^234^U were close to those for ^238^U (Tables 2, 3).

**Table 2:** ^238^U excretion rate (mBq day^−1^) in urine in all age groups

Age group	3–20	21–60	>60
No. of samples	31	36	17
Range	0.44–8.79	0.70–30.54	0.68–6.01
Mean	1.78 ± 1.71	10.12 ± 7.56	2.42 ± 1.63
Median	1.13	9.17	1.63

**Table 3:** ^234^U excretion rate (mBq day^−1^) in urine in all age groups

Age group	3–20	21–60	>60
No. of samples	31	36	17
Range	0.33–9.89	0.90–28.61	0.72–8.42
Mean	2.52 ± 2.41	13.50 ± 8.63	2.71 ± 1.88
Median	1.42	12.88	2.32

**Fig. 4:**
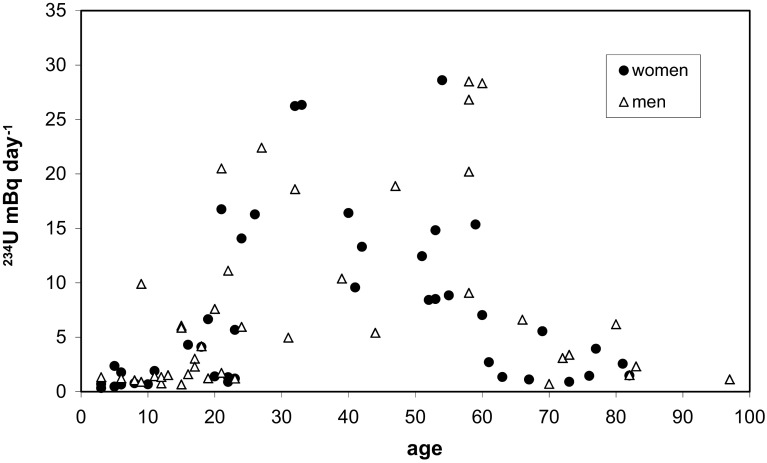
Relationship between daily urinary excretion of ^234^U and age for non-exposed Warsaw citizens

Figure 4 shows ^234^U excretion rate in urine for women and men. The highest value and the average was observed for the age group 21–60 years. For people from 3 to 60 years old values of ^234^U excretion rate increases with age (correlation *R* ^2^ = 0.46). Mean value of all samples was 7.27 ± 7.99 (median 4.03) mBq day^−1^.

## Conclusions

The average value of ^238^U excretion rate 5.49 mBq day^−1^ is higher than that published for non-exposed people, from Germany (0.18–0.86 mBq day^−1^) [4] and India (average 0.15 mBq day^−1^) [7] but in a good agreement with data from Jordania (average 3.95 mBq day^−1^) [6] and those given in ICRP No. 23 (0.6–6 mBq day^−1^) [1]. The excretion rate of ^238^U and ^234^U was found to increase with age up to 60 years. For older people slightly decrease in uranium concentration is observed. Total values of ^238^U 5.49 and ^234^U 7.27 mBq day ^−1^ and *f*_1_ = 0.02 result in a yearly intake of 100 and 133 Bq respectively. Applying the ingestion dose coefficient of 4.5 × 10^−8^ Sv Bq^−1^ for ^238^U and 4.9 × 10^−8^ Sv Bq^−1^ for ^234^U [8] gives effective internal dose 4.5 and 6.5 μSv year^−1^ respectively. Resulting doses are upon ten times higher than doses given by Pietrzak-Flis et al. (0.36 and 0.48 μSv year^−1^) [2], calculated from dietary intake for central Poland (Warsaw). This may result from a change in dietary habits in recent years and the increased ingestion of mineral and spring waters containing more uranium than tap water.
